# Eukaryotic Translation Initiation Factor 4 Gamma 1 (EIF4G1): a target for cancer therapeutic intervention?

**DOI:** 10.1186/s12935-019-0947-2

**Published:** 2019-08-31

**Authors:** Praveen Kumar Jaiswal, Sweaty Koul, Nallasivam Palanisamy, Hari K. Koul

**Affiliations:** 1 Department of Biochemistry and Molecular Biology, LSU Health Sciences Center, 1501 Kings Highway, PO Box 33932, Shreveport, LA 71130-3932 USA; 2 Department of Urology, LSU Health Sciences Center Shreveport, Shreveport, LA 71130 USA; 3 Feist Weiller Cancer Center, LSU Health Sciences Center Shreveport, Shreveport, LA 71130 USA; 4 Department of Urology, Henry Ford Health System, Vattikuti Urology Institute, One Ford Place 2D26, Detroit, MI 48202 USA

**Keywords:** Cap-dependent translation, EIF4G1, TMA, TCGA, DepMap, EIF4G complex inhibitor

## Abstract

**Background:**

Cap-dependent mRNA translation is essential for the translation of key oncogenic proteins at optimal levels and is highly regulated by the rate limiting, initiation step in protein synthesis. Eukaryotic Translation Initiation Factor 4 Gamma 1 (EIF4G1) serves as a scaffold for assembly of cap-dependent translation components in EIF4F complex formation. In the current study, we analyzed the role and expression of EIF4G1 in Pan human cancer panels through various approaches.

**Methods:**

Immunohistochemistry analysis of EIF4G1 protein was done on high-density multi-organ Human Cancer tissue microarray (TMA) derived from the patient samples from different cancers. We used multiple clinical cohorts to analyze the EIF4G1 mRNA expression across human cancers. TCGA data analysis of EIF4G1 was done through Ualcan and c-bioportal web servers. Western blots for EIF4G1 protein was done for different cell lines in representing the multiple cancer types. Dependency score was calculated through Cancer Dependency Map. Clonogenic, tumorosphere assay and cell invasion assay were done with EIF4G complex inhibitor. Association of EIF4G1 mRNA and Kaplan–Meier survival analysis was done on available TCGA datasets.

**Results:**

We observed an increase in EIF4G1 protein levels in tissue sections from different cancers as compared to their respective normal tissue. Our analysis of the TCGA data revealed that EIF4G1 mRNA expression is significantly increased in tumor tissues compared to respective control tissues across human cancers and variable expression was observed among different datasets. We discovered that alteration frequency in EIF4G1 is prevalent in human cancers e.g. prostate cancer (~ 25%), ovarian cancer (~ 15%), Head and Neck cancer (~ 13%) and cervical cancer (~ 12.5%). EIF4G1 mRNA and protein levels were high across cancer cell lines from multiple organs. Our analysis of DepMap datasets utilizing depletion assays revealed that EIF4G1 is critical for cancer cell survival. Treatment with EIF4G complex inhibitor impaired clonogenic, tumorosphere formation potential and inhibited cell invasion. Moreover, higher EIF4G1 mRNA level was associated with a lower median survival of patients in multiple tumor types.

**Conclusions:**

These studies show that EIF4G1 is amplified/over-expressed in multiple cancers and plays an essential role in cancer cell survival, as such EIF4G1 could serve as a novel potential target for therapeutic intervention across many cancers.

## Background

Regulation of gene expression is in-part governed by translating the specific messenger RNAs (mRNA) into a functional protein. Rapidly dividing cancer cells require high protein turnover content [1] for their maintenance and other physiological functions. Translation is the most energy consuming affair [2] compared to transcription and replication. Cancer cells require tightly controlled gene expression, which is in-part dependent on translational process, as such cancer cells upregulate translational machinery to maintain the high concentration of addictive oncogenes. Protein translation is carried out by the complex machinery and is highly regulated processes, which involves initiation, elongation, termination and ribosome recycling [3].

Initiation of translation can be cap-dependent or cap-independent. To meet the criteria for enormous protein synthesis and mRNAs translation of different oncogenes, cancer cells rely on cap-dependent translation. Cap-dependent translation initiation starts at 5′cap (m7GTP) moiety of eukaryotic initiation factor 4 F (EIF4F) complex which has the cap-binding protein EIF4E, EIF4A (helicase) and EIF4G (scaffolding protein). Translational regulation is done majorly at the initiation step and is highly regulated by the different component of EIF4F complex [4, 5]. The EIF4F complex recruits ribosomes to mRNA such that the 5′ untranslated region (5′ UTR) can be scanned by ribosomes in search of an initiation codon [5]. EIF4G serves as a scaffold for the assembly of the translation initiation complex to initiate translation machinery at the 40S ribosome binding to the mRNA. They recruit 40S ribosome to mRNA by 43S initiation complex formation [6].

An interaction of EIF4G and EIF4E are critical for the formation of the EIF4F complex and initiation of cap-dependent translation [7]. The cap at the 5′ terminus of a mRNA and the poly(A) tail is required for maximal mRNA translational efficiency [8]. Majority of eukaryotic mRNAs, except histone mRNAs, comprised of a poly(A) (polyadenylated) tail at 3′ end. Poly(A) binding protein (PABPs) interact EIF4G, thus circularization of the mRNA [9, 10] that improves the translation efficiency by facilitating the use or recycling of 40S ribosomes [11]. The activity of EIF4F is regulated by two major signaling networks PI3K/mTOR and RAS/MAPK (mitogen-activated protein kinase) pathway [12, 13].

Cancer cells depend on the activation of oncogenic signaling/pathways for their survival, a phenomenon known as “oncogene addiction” [14]. Cellular transformation and tumor development promoted by different oncogenic pathways/signaling such as PI3K, Myc, and Ras were orchestrated by deregulated translation control in cancer cells [1, 15, 16]. The PI3K-AKT-mTOR pathway is one of the most commonly altered pathways in cancer and has a role in promoting translation initiation through mTORC1-dependent hyper-activation of EIF4E [17–19]. Collectively, these oncogenic pathways and signaling regulate the global changes in protein synthesis and regulation/expression of selective mRNA translation of specific mRNAs.

EIF4G family comprises three isoforms EIF4G1, EIF4G2 and EIF4G3 [20]. Isoform EIF4G1 and EIF4G3 are required for the cap-dependent translation, however, EIF4G2 isoform is involved in IRES-dependent translation in cells [20, 21]. Among all the isoforms EIF4G1 is the major isoform (> 85%) [22] and is critical for cap-dependent translation. EIF4G1 provides a platform for assembly of components of EIF4F complex such as EIF4E and EIF4A and other components that start cap-dependent translation at m7-GTP cap moiety. We recently observed that EIF4G1 is overexpressed/amplified in the majority of patients with castration-resistant prostate cancer (CRPC), and plays an essential role in prostate cancer progression, cell growth and metastasis [23]. Here in the present study, we analyzed the expression and role of EIF4G1 across pan-human cancers. Our analysis of the TCGA data by using Ualcan and c-bioportal server revealed the higher expression of EIF4G1 mRNA expression across human cancers. Moreover, increased expression of EIF4G1 was associated with a lower median survival of patients with multiple cancers. This is the first study demonstrating overexpression/amplification of EIF4G1 across pan-cancers, highlighting a broader role for EIF4G1. These results suggest EIF4G1 may serve as a novel target for therapy in multiple malignancies. However further studies are required to develop these concepts and test the usefulness of targeting EIF4G1 in vivo using preclinical model systems.

## Material and methods

### Materials

Antibody for EIF4G1 (Cell Signaling #2858, 1:1000 dilution) and GAPDH (Sigma G8795, 1:3000 dilution) were used to probe respective proteins of interest in western blot and IHC (1:50 dilution for EIF4G1).

### Cell lines and culture

We used several cancer cell lines representing different cancers viz. head and neck cancer (FaDU, PCI-13), pancreatic cancer (Mia PaCa-2, BxPC3), lung cancer (H1975, HCC827), renal cancer (A498, UOK262), breast cancer (MB231, MCF-7), bladder cancer (HTB9, HTB5), prostate cancer (LNCaP, C4-2B) and cervical cancer (HeLA). Cells were cultured as described per ATCC protocol.

### Data mining from TCGA datasets

Genetic alteration in EIF4G1 such as mutation, fusion, amplification, and deep deletions analysis were done from TCGA (The Cancer Genome Atlas) data sets for different cancers through UALCAN (http://ualcan.path.uab.edu/) [24] web server. Further, the data mining for EIF4G1 mRNA expression on primary tumors of respective cancers and normal samples were also done from TCGA data sets through UALCAN web server. We also did the survival analysis based on EIF4G1 expression for different cancers from available TCGA datasets. For gene expression changes for EIF4G1 from the different clinical datasets, we used the cBioPortal (http://www.cbioportal.org) tool [25, 26] and UALCAN server. Genetic alterations in EIF4G1 such as mRNA up-regulation, amplification, deep deletion, and down-regulation were assessed across pan-cancers.

### Tissue microarrays and immunohistochemistry (IHC)

High-density multiple organ human tissue microarray (TMA) derived from different human cancers were purchased from US Biomax, Inc.(Cat#MC5003c) and were used to test the EIF4G1 protein levels in the patient tissues and respective normal tissues. This High-density TMA containing 20 types of organs (20 cases of carcinoma and 5 normal tissues per organ), a single core per case. Immunohistochemistry for EIF4G1 on this multiorgan TMA was performed as described [27]. The TMA was scanned through Leica Biosystems CS2 scanner. Aperio ImageScope viewer was used to take photomicrographs for representative tissue sections.

### Western blot

Western blot for EIF4G1 was performed as described [28] for different cell lines. Blots were scanned for the EIF4G1 protein and GAPDH as a loading control by LI-COR Odyssey CLx (LI-COR, Lincoln, USA) system by using IRDye 680 goat anti-mouse/IRDye 800 goat anti-rabbit secondary antibodies as described [23].

### Cancer dependency map for EIF4G1

Cancer dependency map for EIF4G1 was developed through DepMap portal developed by Broad Institute [29] for the pan-cancers.

### Clonogenic and tumorosphere formation assay

Clonogenic activity was measured as described [30] and tumor sphere formation was done as described [23]. The tumorosphere area was measured by Image studio 5.2 software available by LI-COR Odyssey system.

### Cell invasion assay

Trans-well cell invasion assay was performed in the presence and absence of 4EGI-1, a small molecule inhibitor of EIF4G-EIF4E complex and the results were analyzed as described previously [31].

### Statistical analysis

Data are expressed as mean ± standard deviations (SD). The two-tailed Student *t* test was used for statistical analysis and GraphPad Prism5 was used for statistical analysis. Significant differences in p-values are shown as * < 0.05, ** < 0.01.

## Results

### High protein levels of EIF4G1 were observed in the tissue sections from different cancers

**Fig. 1:**
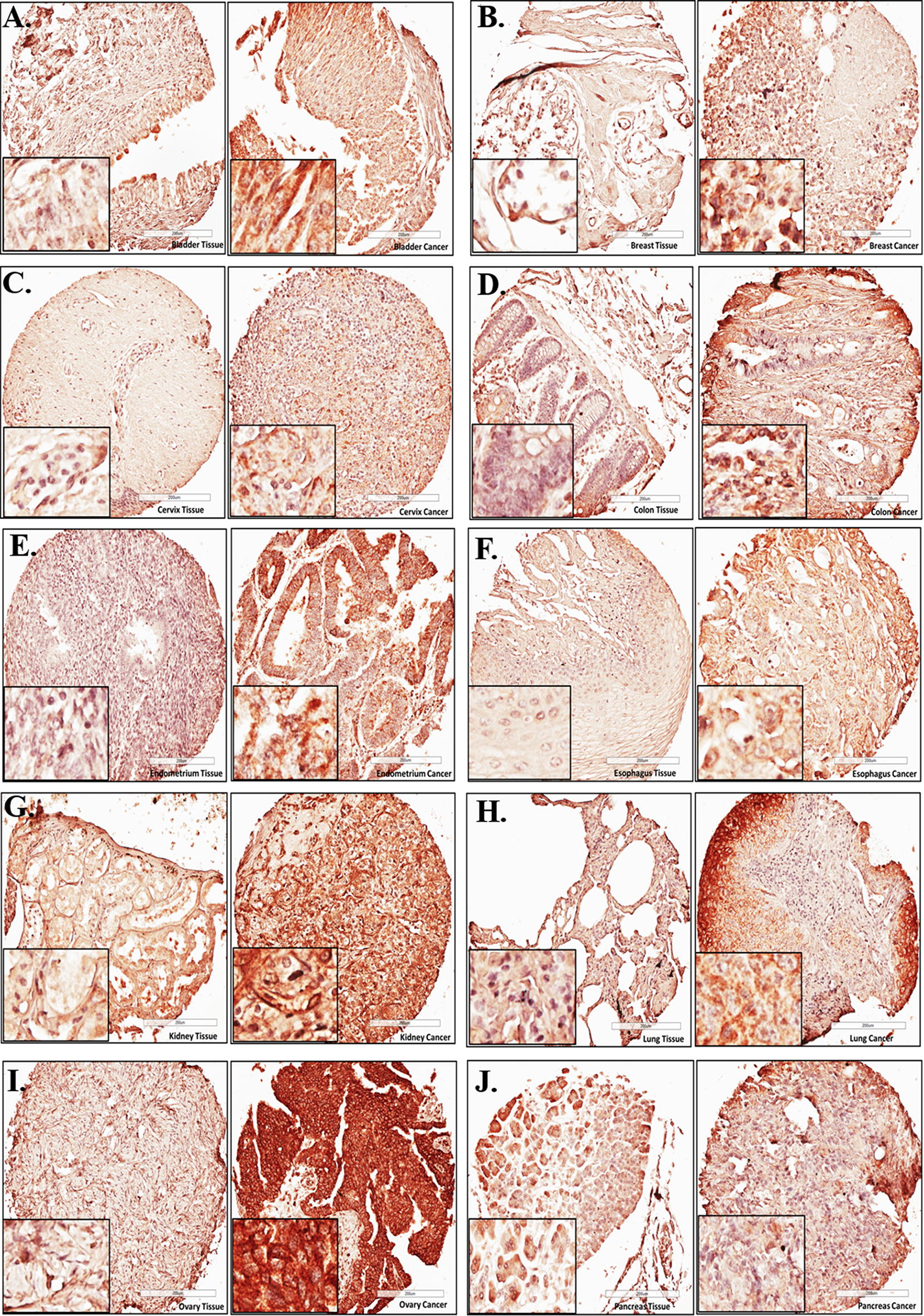
High protein levels of EIF4G1 were observed in the tissue sections from different cancers: Representative photomicrographs for EIF4G1 IHC for **A** bladder, **B** breast, **C** cervical, **D** colon, **E** endometrial, **F** esophagus, **G** kidney, **H** lung, **I** ovarian, **j** pancreatic cancer patients (right side) with respective normal tissues (left side). High-density TMA was scanned through Leica Biosystems CS2 scanner. Aperio ImageScope viewer was used to take photomicrographs for representative tissue sections of IHC

Examination of EIF4G1 protein in the tissue sections by IHC, revealed an increase in EIF4G1 protein levels in cancer tissues (right side) derived from different organs viz. bladder, breast, cervical, colon, endometrium, esophagus, kidney, lung, ovary, pancreas (Fig. 1A–J), head and neck, stomach and testis cancer (Additional file 1: Figure S1A–C) as compared to their respective normal tissues (left side).

### Cancer tissue expresses higher levels of EIF4G1 mRNA

**Fig. 2:**
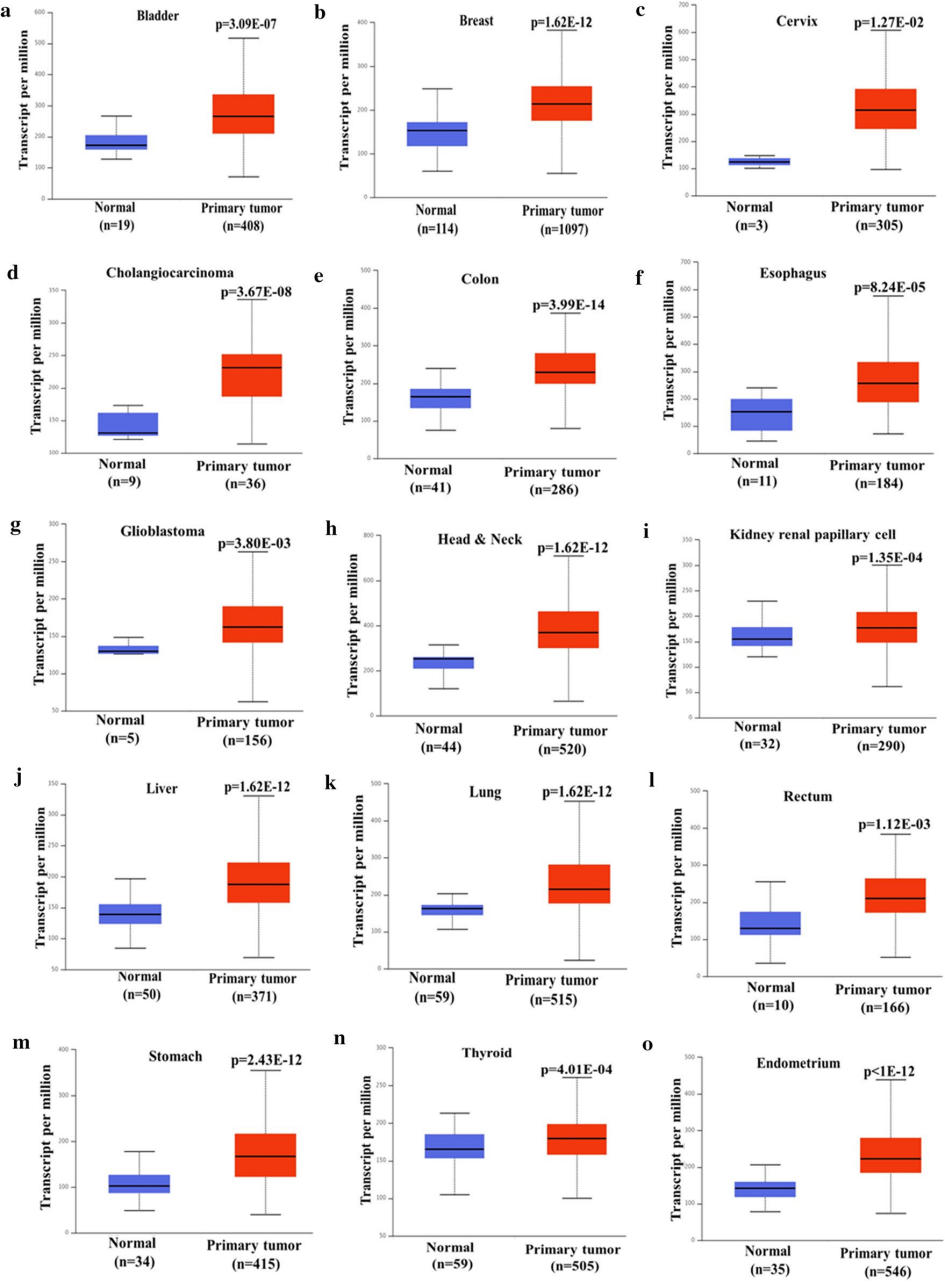
Cancer tissue expresses higher levels of EIF4G1 mRNA: EIF4G1 mRNA data were extracted from TCGA datasets through UALCAN web server. We observed elevated mRNA level of EIF4G1 in primary tumor as compared to normal tissue for **a** bladder, **b** breast, **c** cervix, **d** cholangiocarcinoma, **e** colon, **f** esophagus, **g** glioblastoma, **h** head and neck, **i** kidney renal papillary cell, **j** liver, **k** lung, **l** rectum, **m** stomach, **n** thyroid, **o** endometrial cancer

We analyzed mRNA expression data for EIF4G1 from TCGA datasets through UALCAN web server, samples from different cancers, which includes primary tumor and normal tissue of bladder, breast, cervix, cholangiocarcinoma, colon, esophagus, glioblastoma, head and neck, kidney renal papillary cell, liver, lung, prostate, rectum, stomach, thyroid and endometrium. Our results showed that mRNA level of EIF4G1 in primary tumor was universally high compared to normal tissue of bladder (p = 3.09E−07), breast (p = 1.62E−12), cervix (p = 1.27E−2), cholangiocarcinoma (p = 3.67E−08), colon (p = 3.99E−14), esophagus (p = 8.24E−05), glioblastoma (p = 3.80E−03), head and neck (p = 1.62E−12), kidney renal papillary cell (p = 1.35E−04), liver (p = 1.62E−12), lung (p = 1.62E−12), rectum (p = 1.12E−03), stomach (p = 2.43E−12), thyroid (p = 4.01E−04) and endometrial (p < 1E−12) cancer (Fig. 2a–o). Overall, data showed overexpression of EIF4G1 mRNA across human cancers that is in-line with the findings from IHC data for EIF4G1.

### Cancer tissues invariably express high levels of EIF4G1 as compared to normal tissues

**Fig. 3:**
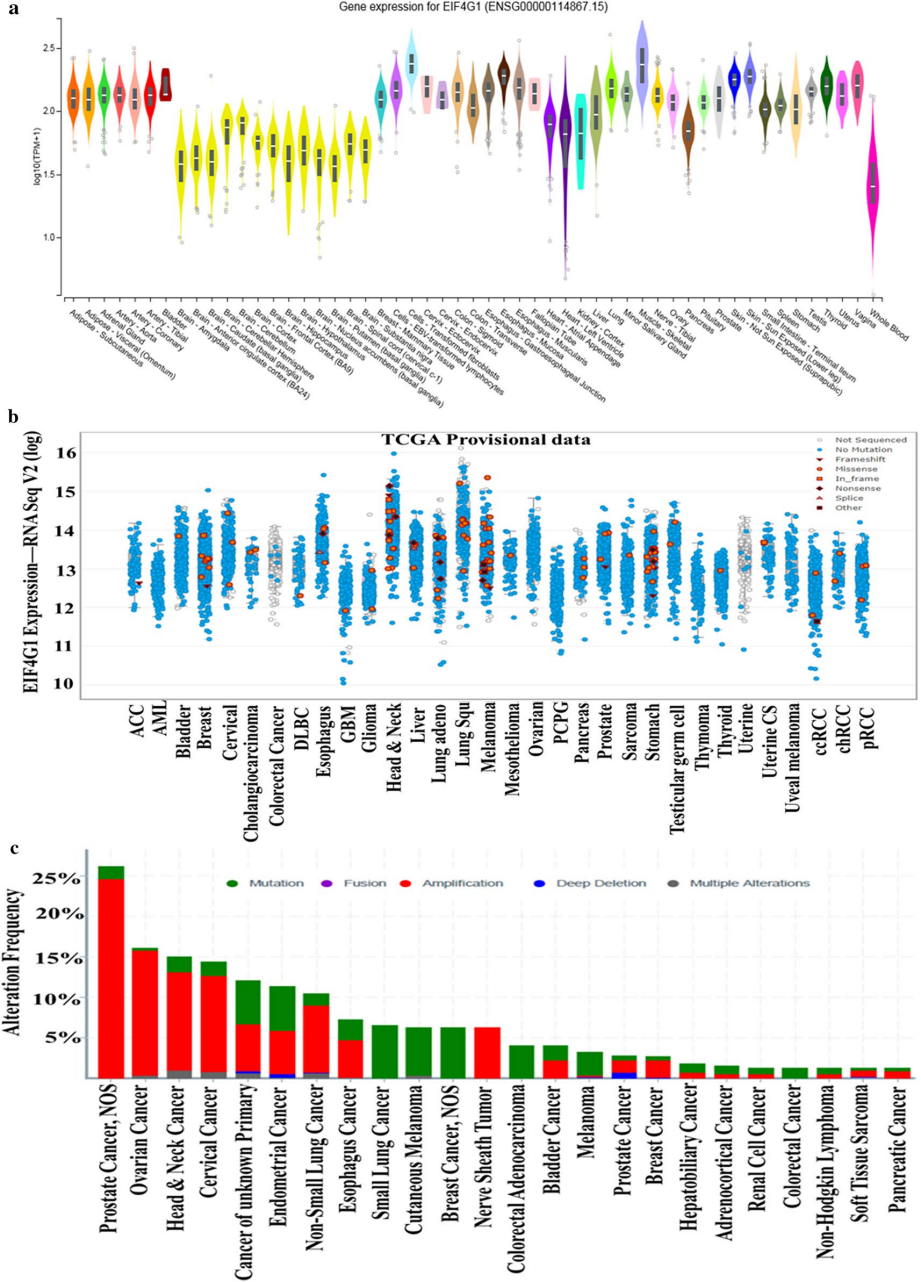
Cancer tissues invariably express high levels of EIF4G1 as compared to normal tissues: **a** Violin plot showed mRNA data for EIF4G1 on non-disease tissues from multiple organs, the analysis was done through the GTEx portal. Expression values are shown in log TPM (Transcripts per Million). **b** mRNA data for EIF4G1 in TCGA provisional dataset for different cancers with different mutational load in EIF4G1, the analysis was done through cBioPortal web-server. **c** Percent alteration frequency changes in EIF4G1 based on mutation, fusion, amplification, deep deletion, and multiple alternations across human cancers. The analysis was done on TCGA datasets through cBioPortal for alteration frequency of EIF4G1

We analyzed the mRNA expression of EIF4G1 in normal non-disease tissues from multiple organs samples through the GTEx (Genotype-Tissue Expression project) portal. This portal has mRNA expression data for non-disease, normal tissues from different organs. We analyze the EIF4G1 mRNA expression data and found a lower levels of EIF4G1 expression across normal tissues derived from different organs (Fig. 3a) as compared to mRNA expression data for EIF4G1 in cancer tissues from pan-cancers (Fig. 3b). We further analyzed that EIF4G1 mRNA expression in provisional TCGA datasets across multiple cancer types with the different mutational patterns (Fig. 3b). We found an elevated level of EIF4G1 (6 to 11 fold increase) across human cancers as compared to normal tissue samples. We next analyzed the data through cBioPortal for alteration frequency of EIF4G1 based on mutation, fusion, amplification, deep deletion and multiple alternations across human cancer. We observed that amplification of EIF4G1 were prominent in prostate cancer (~ 25%), ovarian cancer (~ 15%), Head and neck cancer (~ 13%) and cervical cancer (~ 12.5%). However, percent amplification of EIF4G1 varies for other cancers from ~ 5% (Endometrial cancer), ~ 9% (non-small lung cancer) and ~ 4% (Esophagus cancer). Mutation pattern in EIF4G1 in patients with Small lung cancer, cutaneous melanoma, and Breast cancer showed ~ 7.5% alteration frequency (Fig. 3c). Altogether, data suggested that there is a high expression of EIF4G1 mRNA levels across human pan-cancers.

### EIF4G1, amplification and mRNA up-regulation were observed across human cancers

**Fig. 4:**
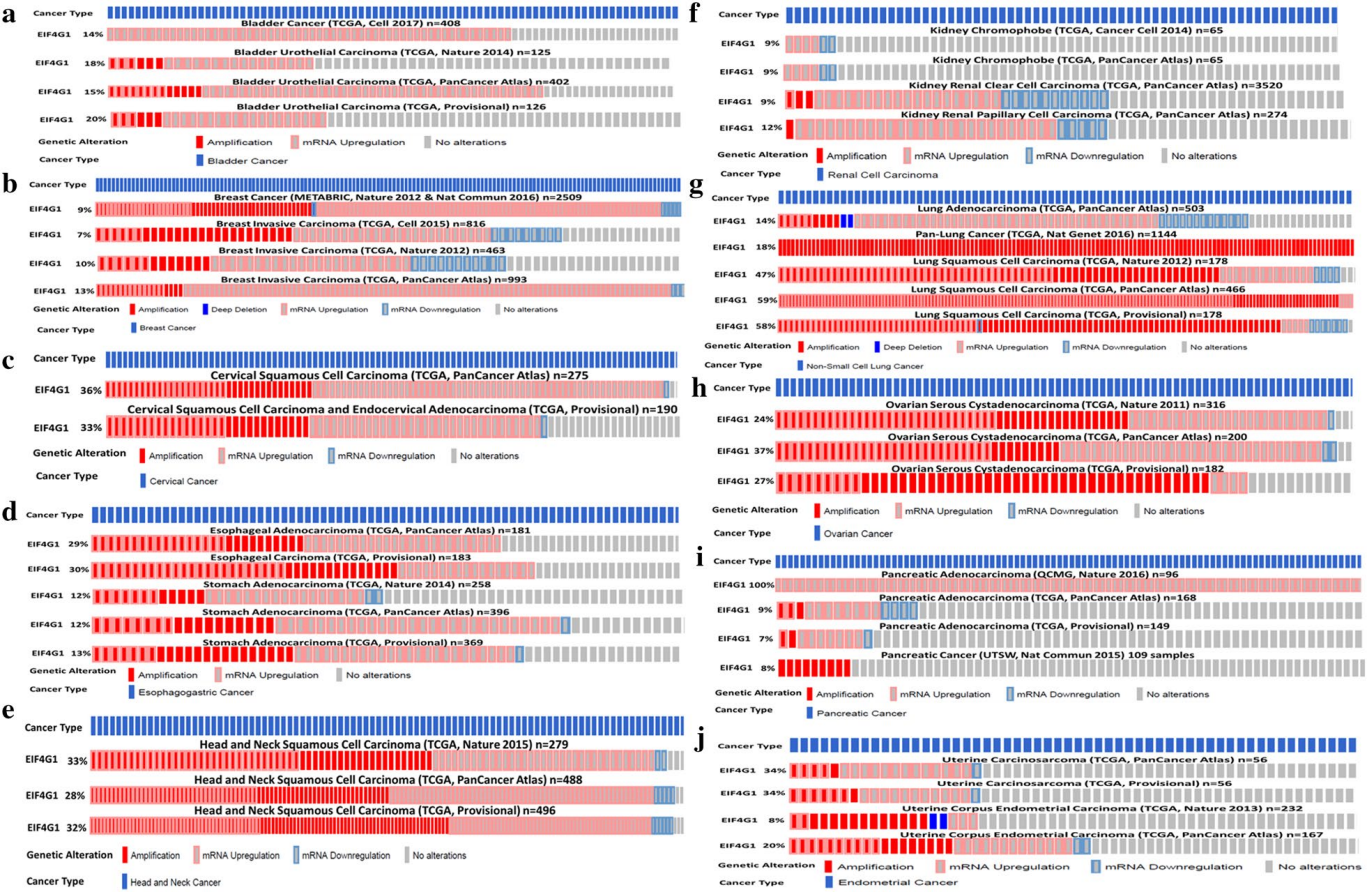
EIF4G1, amplification and mRNA up-regulation was observed across human Cancers: EIF4G1, amplification, and mRNA up-regulation analysis was done on TCGA dataset through c-BioPortal. Genetic alteration in EIF4G1 such as amplification and or mRNA upregulation was considered for **a** bladder cancer, **b** breast cancer, **c** cervical cancer, **d** esophageal carcinoma and stomach cancer, **e** head and neck cancer, **f** kidney cancer, **g** lung cancer, **h** ovarian cancer, **i** pancreatic cancer, **j** endometrial cancer patients

We next analyzed the individually available TCGA dataset from different cancers through c-BioPortal web server. Our analysis revealed that 14% to 20% of Bladder cancer patients (n = 126 to n = 408) showed amplification and or up-regulation in EIF4G1 in different datasets (Fig. 4a). With Breast cancer, 9 to 13% of patients (n = 463 to n = 2509) showed amplification and or up-regulation in EIF4G1 (Fig. 4b). Cervical cancer patients (n = 190 to n = 275) showed a range of 33 to 36% amplification and or up-regulation in EIF4G1 (Fig. 4c). Esophageal carcinoma and stomach cancer patients (n = 181 to n = 396) showed 12 to 30% of amplification and or up-regulation in EIF4G1 (Fig. 4d). Further, 28 to 33% of Head and Neck cancer patients (n = 279 to n = 496) showed amplification and or up-regulation in EIF4G1 (Fig. 4e). In case of the patients (n = 65 to n = 3520) with kidney cancer showed 9 to 12% amplification and or up-regulation in EIF4G1 (Fig. 4f). Lung cancer patients (n = 178 to n = 1144) showed a range of 14 to 59% of amplification and or up-regulation in EIF4G1 (Fig. 4g) for different datasets. Ovarian cancer patients (n = 182 to n = 316) showed 24 to 37% of amplification and or up-regulation in EIF4G1 (Fig. 4h). Interestingly, 100% patients (n = 96) of QCMG dataset for Pancreatic cancer showed up-regulation in EIF4G1 (Fig. 4i), another dataset for Pancreatic cancer patients showed a range of 7 to 9% of amplification and or up-regulation in EIF4G1 (Fig. 4i). With Endometrial cancer, 8 to 34% of patients (n = 56 to n = 232) showed amplification and or up-regulation in EIF4G1 (Fig. 4j). In case of adrenal cancer, Brain lower grade glioma, colorectal, liver, and testicular cancer, patients showed a range of 5 to 10% amplification and or up-regulation in EIF4G1 (Additional file 1: Figure S2A–E). Overall, data showed that increased EIF4G1 levels in tumor tissues result from amplification and or mRNA up-regulation.

### Cancer cell lines express higher levels of EIF4G1

**Fig. 5:**
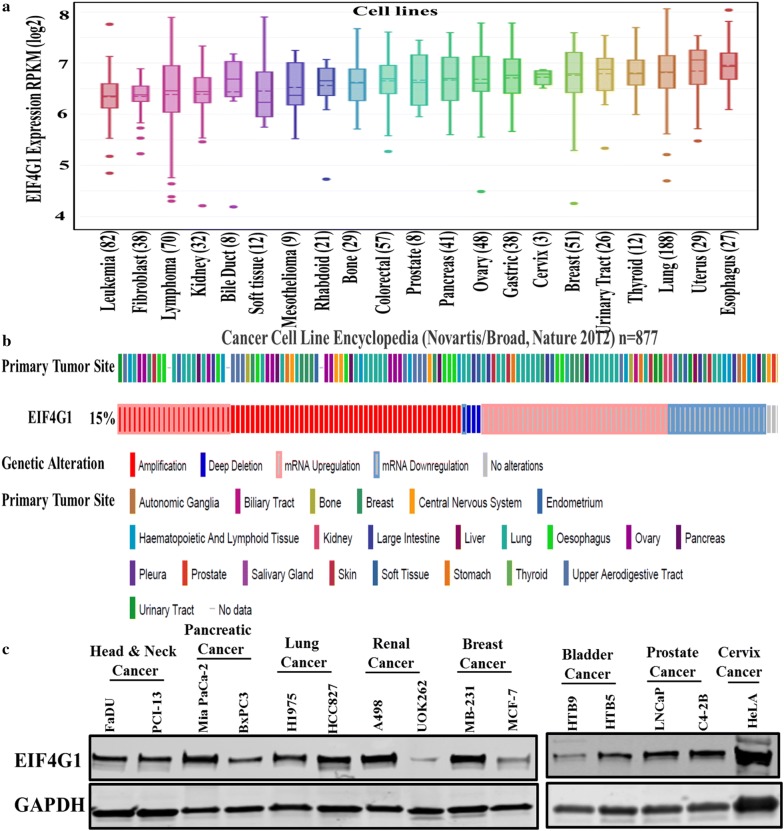
Cancer cell lines express higher levels of EIF4G1: **a** elevated EIF4G1 mRNA levels were observed across the human cancer cell lines derived from different cancers. Data analysis was done through DepMap portal across pan-cancer cell lines. **b** EIF4G1 mRNA expression for different cancer cell lines data available from cancer cell line encyclopedia (CCLE) through c-BioPortal web-server. **c** Representative western blots showing high EIF4G1 protein levels in different cancer cell lines

We analyzed the EIF4G1 mRNA levels across human cancer cell lines derived from different organs through DepMap portal and found an elevated expression in EIF4G1 across different human cancer cell lines (Fig. 5a). Next, we analyzed the EIF4G1 mRNA expression data available from the cancer cell line encyclopedia (CCLE) through c-BioPortal and found that 15% of the cancer cell lines originated from different organs have genetic alteration as measured by amplification, mRNA upregulation and deep deletion in EIF4G1 (Fig. 5b). We also analyzed the EIF4G1 protein level by western blot in the multiple human cancer cell lines viz. from head and neck, pancreatic, lung, renal, breast, bladder, prostate, and cervical cancer and found a high level of EIF4G1 protein level in these cell lines (Fig. 5c).

### EIF4G1 is critical for cancer cell survival

**Fig. 6:**
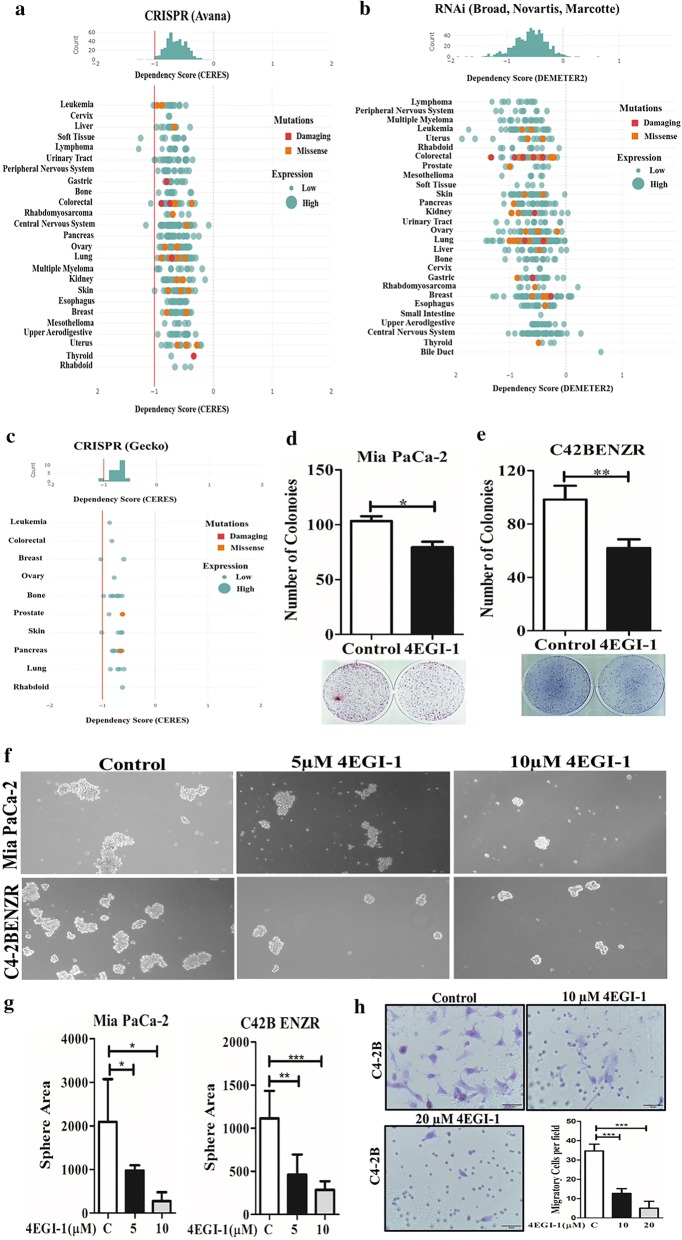
Critical requirement of EIF4G1 for cancer cell survival: Cancer cell survival analysis was done through DepMap portal. **a** CERES dependency score is based on data from a depletion assay for EIF4G1 by CRISPR-Cas9 (Avana) across cancers. **b** DEMETER2 score is based on shRNA (or siRNA) library containing multiple reagents designed to target the EIF4G1 gene in combined RNAi from Broad, Novartis, and Marcortte datasets. **c** CERES dependency score is based on data from a depletion assay for EIF4G1 by CRISPR-Cas9 (Gecko) across cancers. **d**, **e** Colony formation for Mia PaCa-2 (**d**) and C4-2BENZR (**e**) cells with control and treatment with EIF4G-EIF4E complex inhibitor (4EGI-1, 10 µM). The Colonies were counted using an NIH ImageJ software. Error bar represents ± SD. **f** Treatment with 4EGI-1 inhibitor (5/10 µM) significantly impaired the tumorosphere formation in for Mia PaCa-2 and C4-2BENZR cell lines. **g** The tumorosphere area was reduced significantly by treatment with 4EGI-1 inhibitor (5/10 µM). Error bar represents ± SD. **h** Representative Image (×40) and graph of Trans-well cell invasion assays on C4-2B cells. Treatment with 4EGI-1 inhibitor (10/20 µM) for 48 h, significantly inhibited cell invasion. Error bar represents ± SD

We analyzed the requirement of EIF4G1 on cancer cell survival through DepMap portal. Results of genome-wide CRISPR-Cas9 screens with the Avana sgRNA library in 341 cancer cell lines showed EIF4G1 dependency across human cancers. The CERES dependency score is based on data from the depletion assay by CRISPR-Cas9 system. A lower CERES score shows a higher probability that the EIF4G1 is essential for the given cell lines. A score of 0, shows a gene is not essential and likewise − 1 score showed a median of all pan essential genes required for cancer cell survival (red line) (Fig. 6a). Data analysis of EIF4G1 and dependency on cancer cell survival, we analyzed the dataset through DepMap based on shRNA (or siRNA) inhibition for EIF4G1 in combined RNAi from Broad, Novartis and Marcortte datasets. DEMETER2 score is to infer gene knockdown viability effects (‘‘gene dependency scores’’) for each gene and cell line screened by a shRNA (or siRNA) library containing multiple reagents designed to target the same gene. Lower DEMETER2 scores show more dependency on EIF4G1 across human cancer (Fig. 6b). Data analysis from Genome-scale CRISPR Knock-Out (GeCKO) v2.0 pooled libraries for EIF4G1 showed a similar finding as of Avana CRISPR (Fig. 6c). Summarizing the data based on depletion assay (CRISPR-Cas9, shRNA/siRNA) through DepMap showed the dependency of EIF4G1 for cancer cells survival.

### EIF4G complex inhibitor impairs clonogenic activity, 3D tumorosphere formation and inhibits cell invasion

We further explored the functional role of EIF4G1 in cancer cells, we did the clonogenic and tumorosphere assays with 4EGI-1, a known inhibitor of the EIF4G-EIF4E complex in two model cell lines viz. Mia PaCa-2 and enzalutamide resistance cell lines derived from C4-2B cells (C42B ENZR). Treatment with EIF4G-EIF4E complex inhibitor (4EGI-1) impaired the clonogenic activity (Fig. 6d, e) and 3D-tumorosphere potential (Fig. 6f, g) of these cells. Furthermore, treatment 4EGI-1 inhibited cell invasion (Fig. 6h). Overall, results suggesting the functional role of EIF4G1 in clonogenicity, tumorosphere formation and cell invasion, features associated with tumor progression.

### Increased EIF4G1 mRNA levels were associated with lower median survival in cancer patients

**Fig. 7:**
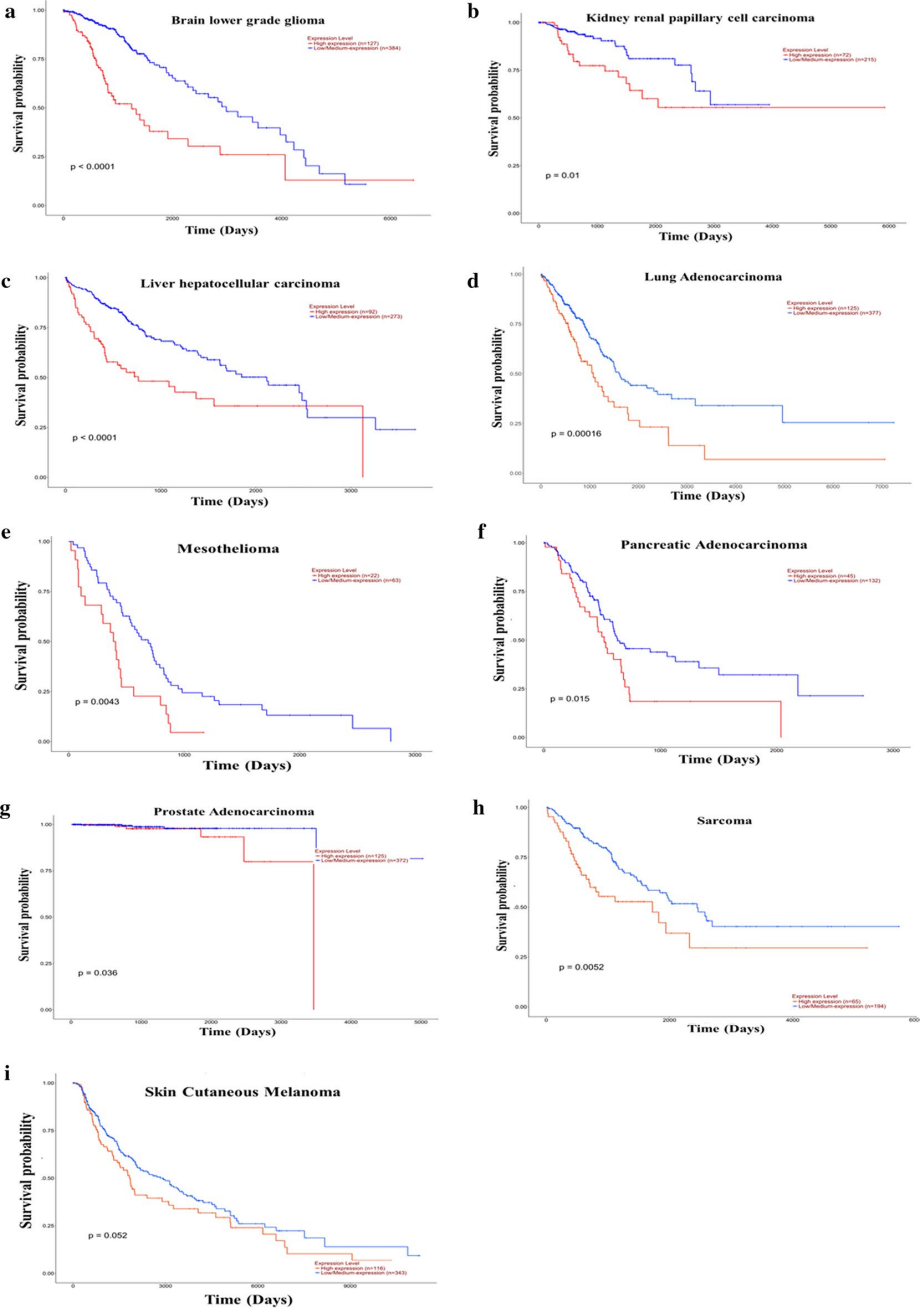
Increased EIF4G1 mRNA expression was associated with lower median survival in cancer patients: Survival probability based on EIF4G1 mRNA expression was performed through UALCAN web server. Analysis of survival data revealed that Brain lower grade glioma, kidney, liver, lung, mesothelioma, pancreatic, prostate cancer, sarcoma, and skin cutaneous melanoma patients with high EIF4G1 expression had lower median survival compared to the patient with low/Medium EIF4G1 expression (**a**–**i**)

We analyzed the survival of patients with different cancer based on mRNA expression data for EIF4G1 from TCGA datasets through UALCAN web server. Our analysis of survival data revealed that Brain lower grade glioma, kidney, liver, lung, mesothelioma, pancreatic, prostate cancer, sarcoma and skin cutaneous melanoma patients with high EIF4G1 expression had significantly (p-value: p < 0.0001, p = 0.01, p < 0.0001, p = 0.00016, p = 0.0043, p = 0.015, p = 0.036, p = 0.0052, p = 0.052 respectively) lower median survival compared to the patient with low/Medium EIF4G1 expression (Fig. 7a–i, Additional file 1: Table S1). Overall, the analysis of survival data revealed that patients with high EIF4G1 expression had lower median survival.

## Discussion

Altered protein synthesis machinery and translational control are the major players in human cancers that are associated with different oncogenic properties that activate the different signaling pathways and selective mRNAs translation and thus contribute to tumor development and disease progression in humans. In the present study, we have aimed to summarize the expression of EIF4G1, a scaffold in the cap-dependent translation initiation machinery across human malignancy that provides the platform for the assembly of EIF4F complex. The PI3K-Akt-mTOR pathway is involved in the mRNA translation and protein synthesis through EIF4F complex which is activated by most known oncogenes [1, 32–35] in human cancers and EIF4G1 is an important component of this complex, which helps in the assembly of other components to start the translation. We recently showed that EIF4G1 is overexpressed/amplified in the majority of patients with castration-resistant prostate cancer (CRPC), and plays an essential role in PCa progression, cell growth and metastasis [23]. It has been shown that EIF4G1 is upregulated in several human cancers [34, 36–42]. We observed an increase in EIF4G1 protein levels in the cancer patient samples that were in-line with EIF4G1 mRNA levels across pan-cancers in TCGA datasets.

The accumulation of genetic alterations induces malignant phenotype [43]. Genomic instability and mutations are enabling characteristics as a hallmark of cancer [44] and tumor progression. Genetic alterations include genetic mutation, gene copy number variation (CNV), gene fusions, gene amplification, loss of heterozygosity (LOH), allelic imbalance (AI) and microsatellite instability (MSI). We found that amplification in EIF4G1 were more prominent in prostate, ovarian, Head and Neck and cervical cancer, while EIF4G1 mutation were more common in patients with small lung cancer, cutaneous melanoma, and Breast cancer, suggesting a distinct pattern of genetic alterations in EIF4G1 across human cancers.

We also analyzed the cancer dependency (cancer cell viability) based on EIF4G1 status across pan-cancer. Cancer dependency interaction map provides a tool to describe the genes imperative for cell viability, thus helps to identify a cancer targets [29]. We analyzed the data through DepMap server for EIF4G1 depletion assay based on CRISPR-Cas9, shRNA/siRNA approach. CERES dependency score method was used for CRISPR-Cas9 based depletion assays (from Avana and Gecko), while accounting for the copy number-specific effects [45] across different cancers. RNAi datasets from Broad, Novartis & Marcotte, DEMETER2 score was considered for calculating the EIF4G1 dependency score. DEMETER score is to infer gene knockdown viability effects screened by shRNA (or siRNA) library containing multiple targets for the same gene. It is possible that cancer dependency of EIF4G1 and cancer cell survival may depend on the fact that cancer cells are fast growing and high expression of EIF4G1 may be required for rapidly dividing cells, however such considerations cannot explain decrease in tumor invasion. We found that EIF4G1 is required for cancer cell proliferation and survival across cancers suggesting the role of EIF4G1 in cancer cell survival across human cancers.

Deregulated oncogenic signaling and pathways that drive the particular phenotype from initiation to development and aggressiveness of cancer, greatly depend on the ability of translational machinery for increased synthesis of oncogenic proteins. In the present study, we have attempted to summarize the expression pattern of EIF4G1, an important component of cap-dependent translation initiation machinery across human malignancy. Thus we posit that therapeutic utility of targeting EIF4G1 is to affect the expression of multiple key oncogenic pathways that are associated with disease progression that rely on cap-dependent translation.

**Fig. 8:**
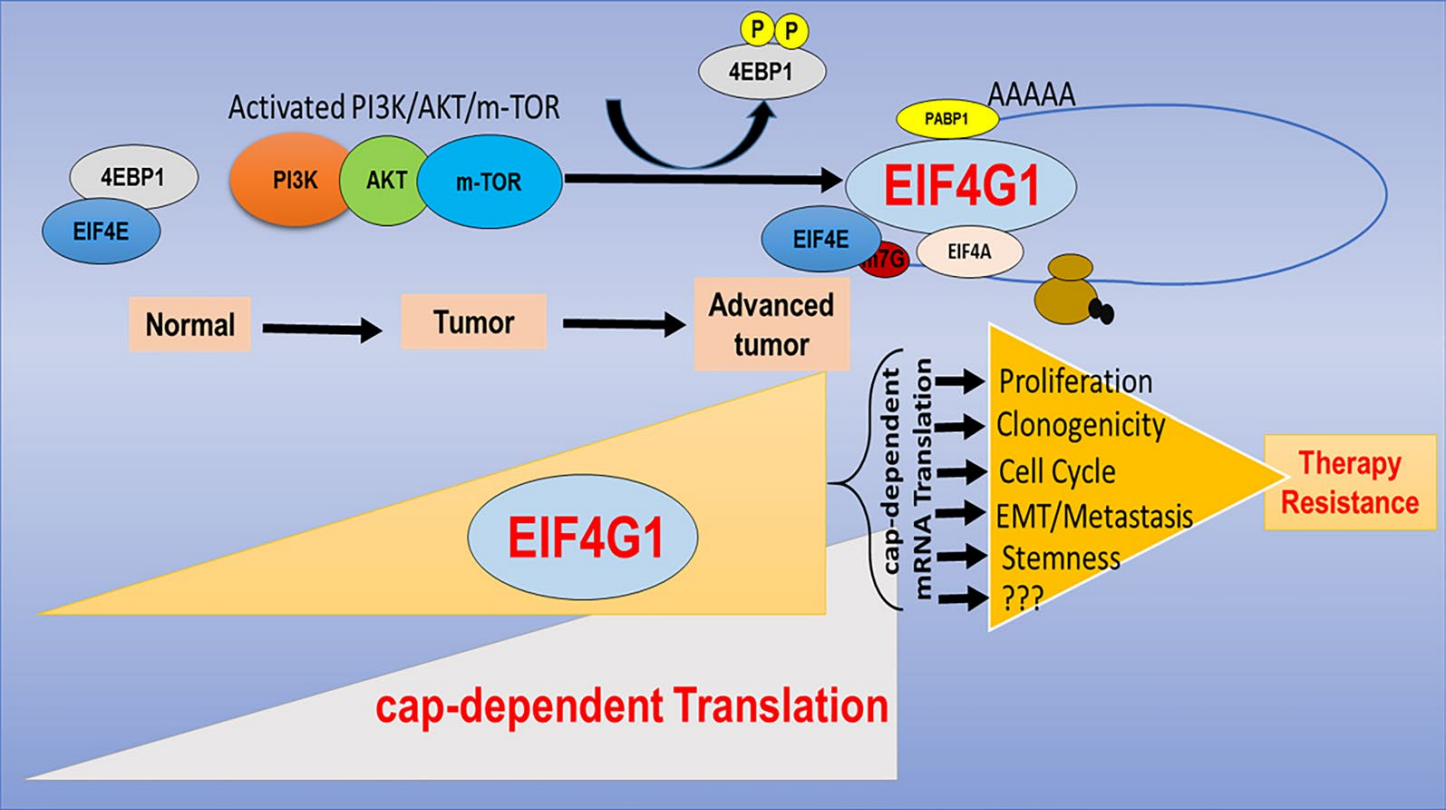
Model for EIF4G1 network in cancers

Based on current understanding of EIF4G1, small molecule inhibitor has been developed for inhibiting EIF4E-EIF4G interaction. Molecules that inhibit the EIF4E–EIF4G interaction such as 4EGI-1 [46] and 4E1RCat [47] are already tested and effective in preclinical models. We tested the functional role of EIF4G1 by treatment with known 4EGI-1 inhibitor and found it impaired the clonogenic and tumorosphere potential in different cancer cell lines resistant to known therapies and also inhibits cancer cell invasion. Patients with high EIF4G1 mRNA levels showed a lower median survival compared to patients with low/medium EIF4G1 expression, suggesting a critical role of EIF4G1 in the patient survival. The basis of the therapeutic window for inhibiting cap-dependent translation at translation initiation step lies for differential expression and requirement of cap-regulated proteins between cancer and non-malignant cells. Targeting cap-dependent translation via EIF4G1 scaffold can disrupt the expression of multiple oncogenic pathways that are associated with tumor progression, offer an excellent target in therapy-resistant cancers (Fig. 8).

## Conclusions

To the best of our knowledge, this is the first comprehensive study showing overexpression/amplification of EIF4G1 across pan-cancers and highlighting a broader role for EIF4G1 across pan-cancers in humans. Our study suggests that EIF4G1 may serve as a novel target for therapy in multiple malignancies. However, further studies are required to develop these concepts and test usefulness of targeting EIF4G1 in cancer using in vivo preclinical model systems, prior to clinical trials in patients.

## Supplementary information

**Additional file 1.** Additional figures and table.

## Data Availability

All data generated or analyzed during this study are included either in this article or in the additional file.

## Abbreviations

EIF4G1: Eukaryotic Translation Initiation Factor 4 Gamma 1
TMA: tissue microarrays
TCGA: The Cancer Genome Atlas
EIF4F: Eukaryotic Translation Initiation Factor 4F
PABP: poly(A) binding protein
MAPK: mitogen-activated protein kinase
CRPC: castration-resistant prostate cancer
