# Supplementary material for: Eukaryotic Translation Initiation Factor 4 Gamma 1 (EIF4G1): a target for cancer therapeutic intervention?
Source: Cancer Cell Int. 2019 Aug 31;19:224. doi: 10.1186/s12935-019-0947-2 (PMC6717390; doi:10.1186/s12935-019-0947-2)
Supplement: Supplementary file 1 — Additional file 1. Additional figures and table. [file 12935_2019_947_MOESM1_ESM.docx]

**Additional figure legends:**

**Additional Figure S1:**

Representative photomicrographs for EIF4G1 IHC for **A.** Head & Neck **B.** Stomach **C.** Testis cancer patients with respective normal tissues.

**
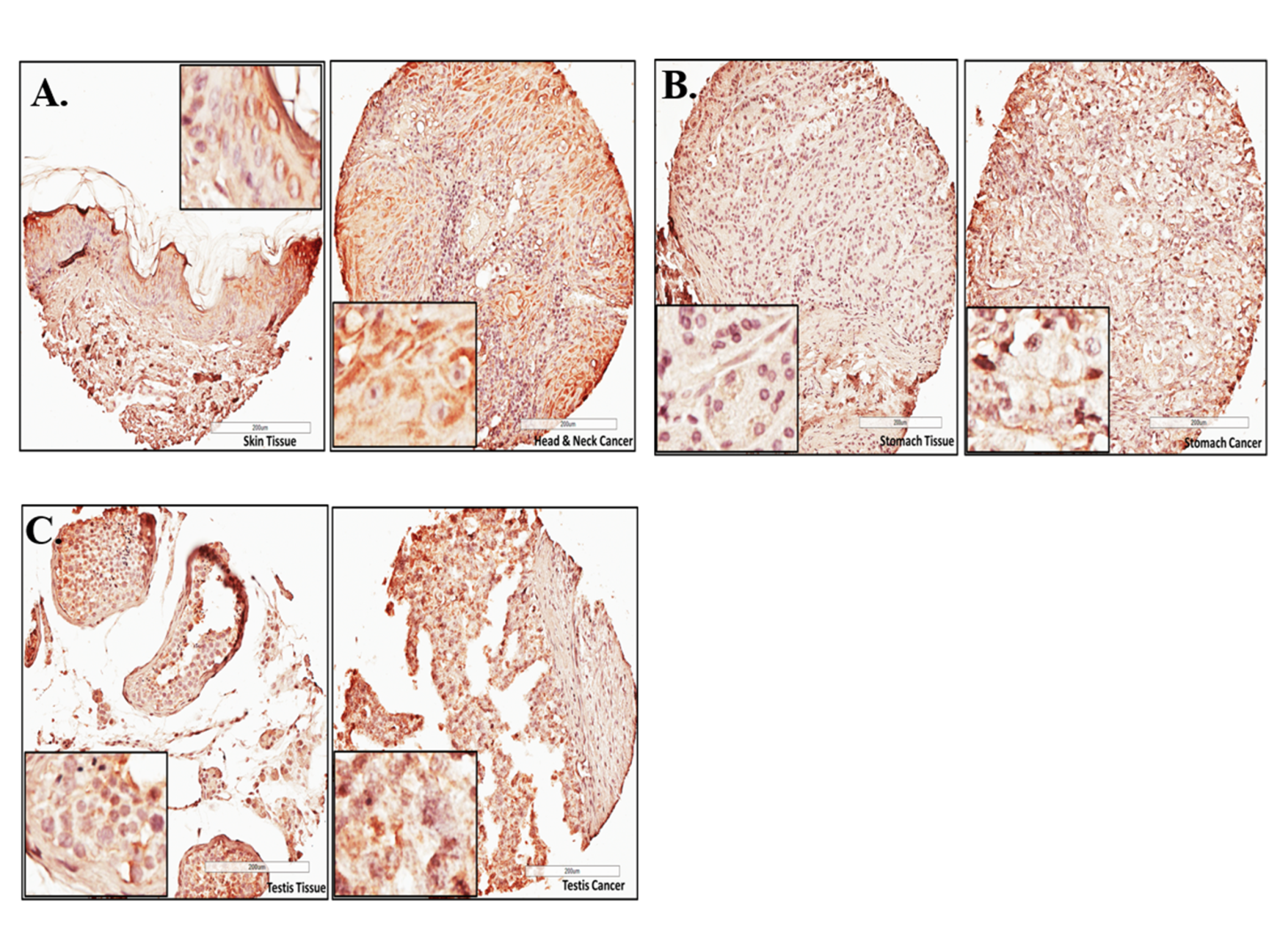
Additional Figure S2:**

EIF4G1, amplification and mRNA up-regulation was observed in adrenal cancer, Brain lower grade glioma, colorectal, liver and testicular cancer patients **(A-E)**.

**
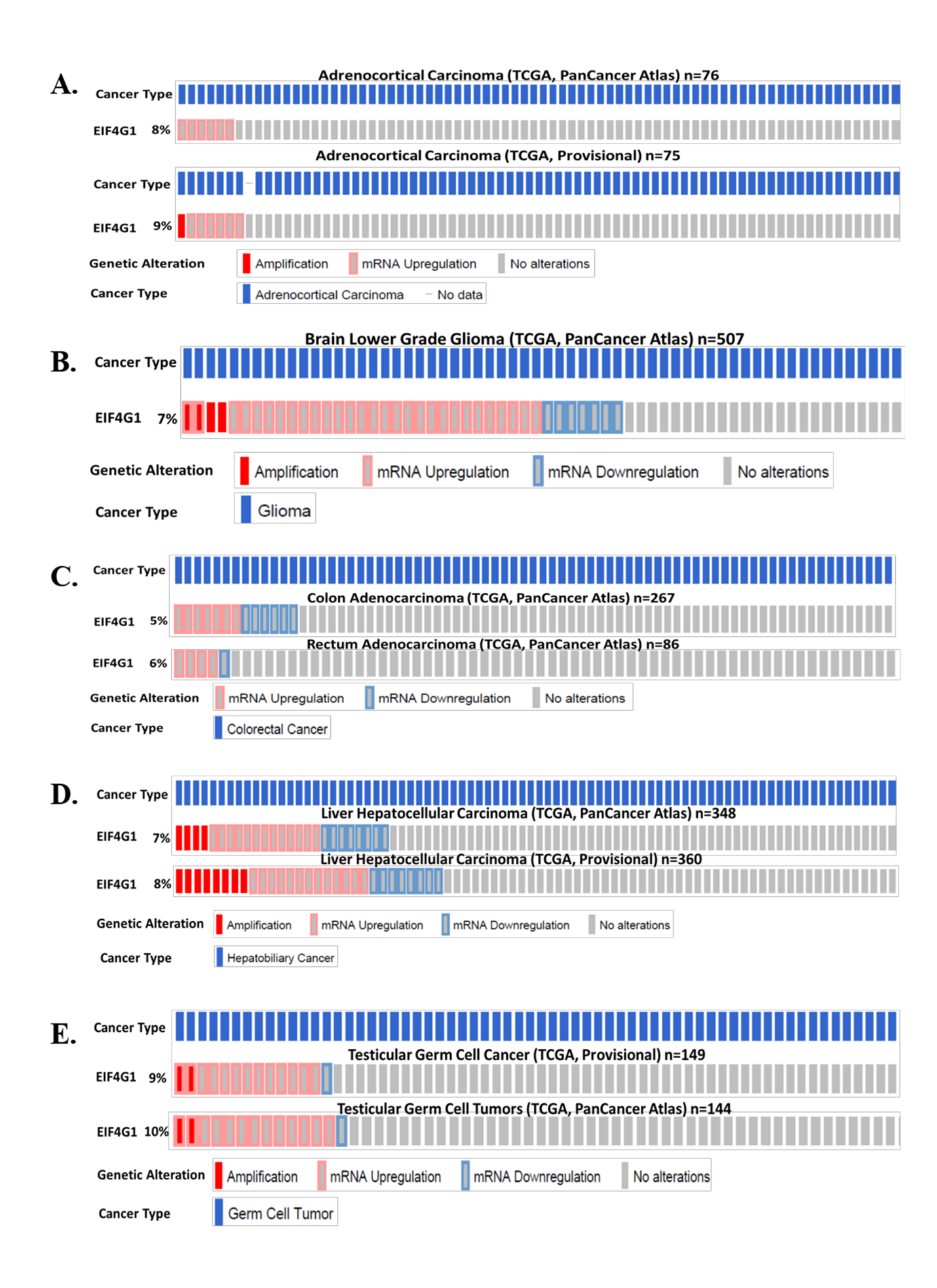
Additional Table S1:**

Median survival in years (approximately) based on high and low/medium EIF4G1 mRNA expression in various cancers.

|  | **Median Survival (≈Years)** | |
| --- | --- | --- |
| **Cancer Type** | **High EIF4G1** | **Low/medium EIF4G1** |
| Brain lower grade glioma | 4.93 | 8.22 |
| Kidney | 6.03 | 8.77 |
| Liver | 2.47 | 4.93 |
| Lung | 3.28 | 4.93 |
| Mesothelioma | 1.36 | 2.47 |
| Pancreatic | 1.92 | 2.33 |
| Prostate | 9.58 | 13.69 |
| Sarcoma | 5.20 | 6.85 |
| Skin cutaneous melanoma | 6.85 | 8.77 |
